# OA07.01. A naturopathic approach to the prevention of cardiovascular disease: a cost effectiveness analysis of a randomized multi-worksite trial

**DOI:** 10.1186/1472-6882-12-S1-O25

**Published:** 2012-06-12

**Authors:** P Herman, O Szczurko, K Cooley, D Seely

**Affiliations:** 1 University of Arizona, Tucson, USA; 2 Canadian College of Naturopathic Medicine and University of Toronto, Toronto, Canada; 3 Canadian College of Naturopathic Medicine, Toronto, Canada

## Purpose

Cardiovascular disease (CVD), a major cause of death and substantial healthcare costs, is also largely preventable. The purpose of this study is to determine the cost-effectiveness of a naturopathic approach to the primary prevention of cardiovascular disease.

## Methods

This study is an economic evaluation alongside a pragmatic, multi-worksite, randomized trial comparing enhanced usual care (EUC; usual care plus biometric screening) to the addition of a naturopathic approach to CVD prevention (NC+EUC; an individualized package of lifestyle counseling and nutritional medicine). Biometric and self-report outcomes were collected at 0, 6, and 12 months. Cost-effectiveness is determined from the societal and employer perspectives for participants who consented (before randomization) to make available their electronic employer sick leave and medical claims data.

## Results

Of the 246 employees who consented to the trial, two-thirds also gave consent to make available their claims and sick leave data: 77 (63.1% of 122) randomized to EUC and 79 (63.7% of 124) randomized to NC+EUC. There were no statistically significant differences between those who did and did not give this consent across baseline characteristics, outcomes, or tendency to miss study visits. After one year NC+EUC resulted in a net decrease of 3.3 (CI: 1.7 -4.8) percentage points in 10-year CVD event risk (NNT=30) and 0.9 (CI: 0.2 – 1.6) points in CVD mortality risk (NNT=111). These risk reductions came with an average net savings of $1138 in societal costs and $1187 in employer costs. There was no change in quality-adjusted life-years across the study year.

## Conclusion

In this multi-worksite-based study, a naturopathic approach to the primary prevention of CVD provided significant reductions in CVD risk over usual care plus biometric screening. These risk reductions are achieved with cost savings to society and employers. Further research into naturopathic approaches to CVD risk reduction is justified.

